# Environmental Disinfection of a Dental Clinic during the Covid-19 Pandemic: A Narrative Insight

**DOI:** 10.1155/2020/8896812

**Published:** 2020-10-28

**Authors:** Antonio Scarano, Francesco Inchingolo, Felice Lorusso

**Affiliations:** ^1^Department of Medical, Oral and Biotechnological Sciences, University of Chieti-Pescara, Via dei Vestini 31, 66100 Chieti, Italy; ^2^Department of Interdisciplinary Medicine, University of Bari “Aldo Moro”, 70121 Bari, Italy

## Abstract

**Background:**

The control of biological hazard risk in health care and dental clinic environments represents a critical point in relation to the Covid-19 infection outbreak and international public health emergency. The purpose of the present review was to evaluate the scientific literature on the no-touch disinfection procedures in dental clinics aiming to limit transmission via airborne particles or fomites using no-touch procedures for environmental decontamination of dental clinics.

**Methods:**

An electronic database literature search was performed to retrieve research papers about Covid-19 and no-touch disinfection topics including full-length articles, editorials, commentaries, and outbreak studies. A total of 86 papers were retrieved by the electronic research.

**Results:**

No clinical article about the decontamination of a dental clinic during the Covid-19 pandemic was detected. About the topic of hospital decontamination, we found different no-touch disinfection procedures used in hospital against highly resistant organisms, but no data were found in the search for such procedures with respect to SARS-CoV-2: (1) aerosolized hydrogen peroxide, (2) H_2_O_2_ vapor, (3) ultraviolet C light, (4) pulsed xenon, and (5) gaseous ozone. One paper was retrieved concerning SARS-CoV-2; 32 documents focused on SARS and MERS. The cleaning and disinfection protocol of health care and dental clinic environment surfaces are essential elements of infection prevention programs, especially during the SARS-CoV-2 pandemic.

**Conclusion:**

The decontamination technique that best suits the needs of the dental clinic is peroxide and hypochlorous which can be sprayed via a device at high turbine speed with the ability of producing small aerosol particles, recommendable also for their low cost.

## 1. Background

A new coronavirus emerged in the central Chinese city of Wuhan in late 2019 [1] and spread rapidly around the world [2] causing the World Health Organization to declare pandemic infection on 11 March 2020 [2]. It is a coronavirus (SARS-CoV-2) that causes pneumonia, moderate to serious respiratory failure, septic shock, and higher risk of death in patients with other pathologies, especially in older people with underlying medical problems like chronic respiratory diseases, cancer, cardiovascular disease, and diabetes [1, 3]. The Covid-19 disease presents nonspecific symptoms such as conjunctivitis, diarrhea, vomiting, shortness of breath, sore throat, fatigue, and muscular pain, and then, there are also asymptomatic patients [4].

This coronavirus pneumonia has a high percentage mortality rate due to risk factors and mortality predictors such as age ≥ 65 years, concomitant cardiovascular pathologies, CD3+CD8+ T cell count ≤ 75 cell·*μ*L^−1^, and cardiac troponin I ≥ 0.05 ng·mL^−1^, [5, 6]. In Italy, the number of confirmed cases was 274.644, including 35.518 deaths as of 4 Sept. 2020, while the great spread of the number of infected cases has caused a lockdown of dental clinical activity and poses a significant risk to personnel dental health care (DHCP) and dental patients. Transmission of SARS-CoV-2 occurs mostly by respiratory droplets over a close distance. It is an aerosol-transmissible disease which can spread when infected people talk, cough, sneeze, or disperse mouth and nasal fomite secretions into the air. Droplets exhaled during speech, sneezes, coughs, and exhalations emit mucosalivary droplets with semiballistic trajectories and a multiphase turbulent gas cloud that entrains ambient air and carries within its clusters of droplets with different droplet sizes. In fact, the exhaled air of infected humans is one of the prime sources of ambient contamination by pathogenic microorganisms. Larger droplets may rapidly settle on the ground or transmit disease to individuals in near proximity, while smaller droplets may remain suspended for a long time and can contribute to disease transmission over great distances [7] and for a long time [8]. Today, there is a worldwide pandemic SARS-CoV-2 agent of serious viral pneumonia in course which is being mitigated by lockdown (quarantine and isolation). The transmission routes of the novel coronavirus include direct transmission (aerosol-transmissible) via droplets that “settle” on another individual, while airborne transmission occurs via small droplets in suspension in the air. In particular, airborne transmission can occur without direct contact and at a long distance via air flows (e.g., if an infected person coughs in a room, leaves, and another person enters) as in the restaurant example presented by Lu et al. [9], while the fomite transmission refers to transmission via droplets (usually larger) that settle on surfaces and are then inoculated by contact of the hands with the contaminated surface which then touch nasal, oral, or eye mucous membranes.

Improving ventilation of health spaces will dilute and clear out potentially infectious aerosols [10, 11].

Viruses or bacteria take flight and remain in the air so that other people can breathe the airborne pathogenic organisms, or these can land on other surfaces. The locally humid and hot atmosphere within the turbulent gas cloud allows the contained droplets to elude evaporation for longer than occurs with isolated droplets [8].

So, for this reason, it is important to implement respiratory infection control with a good prevention strategy in dental practices and health care offices.

In fact, humans have a high-frequency face-touching habit with an average of 23 times in 1 hour, and hands are a common vector for the transmission of health care-associated infections [12]. When air containing pathogenic airborne microorganisms is inhaled by a human, it can cause tuberculosis or Legionella [13], mycoplasma, or influenza, which are great problems in dentistry practice [14]. In dental practices, droplets from infected patients can contaminate the equipment and surfaces with the risk of transferring microorganisms from contaminated surfaces to other patients through hand contact [14–16]. The high-touch equipment surfaces surrounding the patient increase the risk of contamination of these surfaces.

Furthermore, aerosolized virus, fungi, or bacteria in health care facilities can cause infection in the dentistry equipe and all health care workers [17].

So, it is very important that we adopt a proactive infection control approach to sanitation in the dental clinic between one patient and another to minimize the risk of transmission. We can use the disinfection agents through contact, but this procedure is too long and ineffective, because it is impossible to reach all hidden surfaces. The aims of this article are to discuss and suggest some of the novel no-touch disinfection methods in SARS-CoV-2 infection control and prevention of viral transmission in the dental clinic setting, where droplets can be spread by dental tools that aerosolize particles from the mouth, and where surface disinfection is a priority.

In the present review, the scientific literature on the no-touch disinfection procedures in dental clinics aiming to limit transmission via airborne particles or fomites or using no-touch procedures for environmental decontamination of dental clinics was evaluated.

## 2. Methods

A 2-stage procedure was followed. The manuscript included for the evaluation was retrieved from PubMed and MEDLINE, and the data were collected on a specially designed Excel database (Microsoft, Redmond, WA, USA). The database search was performed by two expert reviewers (L.F. and A.S.). Moreover, a second step of the manual search was provided to identify manuscripts eligible for descriptive evaluation. The full text and abstract of the papers included were collected and analyzed. Information available from the literature on the no-touch disinfection of dental clinics in the SARS-CoV-2 pandemic era was acquired. A literature search was also performed to retrieve study articles regarding Covid-19 (SARS-CoV-2) and no-touch disinfection in dental clinics. In the present investigation clinical studies, retrospective and prospective trials and reviews in English full-length articles were included. The exclusion criteria were proceedings, short communications, and letters to the editors.

Data was then selected by focusing on the documentation of the measures of no-touch disinfection, and the actual situation of managing SARS-CoV-2 diffusion in the dental clinic. Also taken into consideration were the articles on the measures implemented in hospitals. The literature search was from database inception up to April 30, 2020. Editorials, commentaries, and outbreak studies were included. Studies in which no-touch disinfection methods were used to evaluate the efficacy of reducing contamination of surfaces were also included.

The Boolean search was performed according to the key words used: “disinfectants AND (Covid-19 OR SARS-CoV-2 infection)”, “no-touch disinfection”, “non-manual disinfection techniques”, “dentistry equipment surface”, “no-touch disinfection AND Covid-19”, “dentistry equipment surface contamination”, “vapor disinfectant AND dental clinic”, and “hospital surfaces contamination and dental clinic contamination”.

## 3. Results

A total of 86 papers were retrieved by the electronic research. No data on the clinical experience in the decontamination of dental clinics during the pandemic of Covid-19 were detected.

We found in literature different no-touch disinfection procedures used in hospitals against highly resistant organisms, but no data was found in the search for such procedures with respect to SARS-CoV-2 (Tables 1–9). Different no-touching disinfection systems have been proposed such as aerosolized hydrogen peroxide [18], hydrogen peroxide-producing systems [19], H_2_O_2_ vapor [20], hydrogen ultraviolet C light [21], pulsed xenon [22], and gaseous ozone [23].

We found more papers on the efficiency of disinfectant agents on other viruses such as severe acute respiratory syndrome (SARS), Middle East Respiratory Syndrome (MERS), mouse hepatitis virus (MHV), canine coronavirus (CCV), and human coronavirus (HCoV).

**Figure 1:**
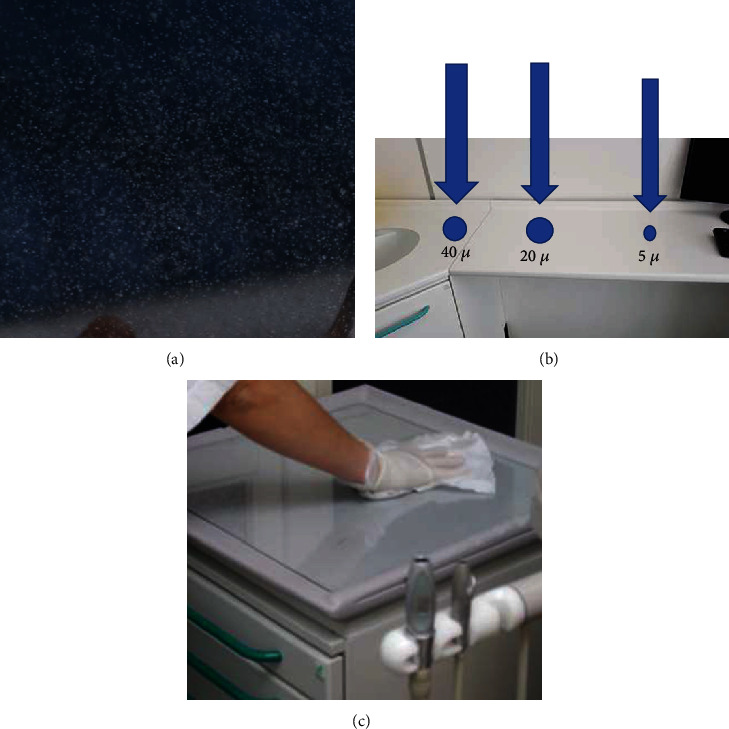
This is a schematic representation as there is no data referenced here in the present paper. (a) Aerosol generating during piezosurgery procedures. (b) Particles of different sizes. Smaller droplets (5 *μ*) may remain suspended for a long time and can settle on all environmental surfaces such as drawer knobs, light handles, unit switches, dental radiograph equipment, reusable containers of dental materials, drawer handles, and dental chairside computers and when these devices are touched, microbial agents can be transferred to other instruments. (c) Manual disinfection of medical device surfaces is very difficult.

**Figure 2:**
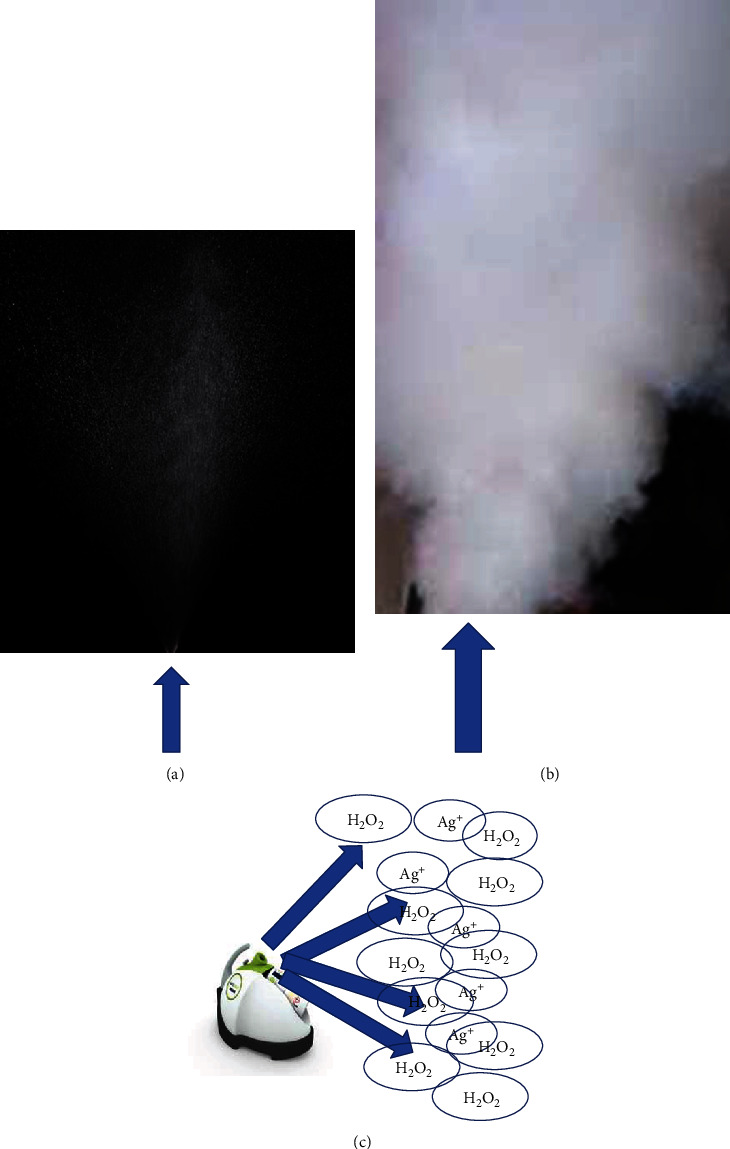
(a) Difference between nebulization. (b) Generator of dilute hydrogen peroxide. (c) Dry fog generated through a turbine at high speed that atomized and sprays disinfectant (arrows).

Aerosolized hydrogen peroxide systems (aHP) generate a dry-mist hydrogen peroxide aerosol of hydrogen peroxide and use a solution containing 5%–7% hydrogen peroxide with or without <50 ppm silver (Nocospay) (Figures 1 and 2) [24, 25]. The generator injects into a room a solution of HP followed by passive aeration and water and is very active against microorganisms.

This device produces a variable particle size of 2–12 *μ*m [26] or of 0.5 *μ*m [27]. Generally, a dosage of 6 mL per m^3^ is recommended which, after erogation, should be left to decompose naturally. This technique uses a low concentration of hydrogen peroxide which, for this reason, metabolically inert spore and catalase-negative bacteria are less susceptible. It is able to reduce contamination of MRSA and C. difficile on work surfaces but has not been shown to eradicate pathogens in clinical practice. It is difficult to achieve the saturation of the environment because aHP is introduced via a unidirectional nozzle by gravity [28].

*H_2_O_2_ vapor heat-generated vapor*. There are two types of HPV: condensing HPV technology and noncondensing vaporized hydrogen peroxide (VHP) technology. This technology uses a vaporizer heated to 120°C and circulates the HPV through the environmental chamber via a supply and return hose. Condensing systems inject hydrogen peroxide until the air in the room becomes saturated and HP begins to condense on the surfaces. The condensing of HP on surfaces can cause corrosion [29]. The HPV device injects at 2 mL/min for 1, 2, or 5 min followed by 1.5 mL/min for 15 min equating to three different volumes: 25, 27, and 33 mL.

The level of 1 ppm is the max level of exposure according to the Occupational Safety and Health Administration and International Labour Organisation. This procedure requires a first phase injection and second phase aeration for a total time of approximately 2–3 h, varying with the amount of hydrogen peroxide being vaporized.

Noncondensing systems produce dry gas by a vaporized hydrogen peroxide system that utilizes erogation of 30%–35% aqueous hydrogen/peroxide (VHP) at high-velocity air. VHP systems have a generator which delivers until the air in the enclosure becomes saturated and hydrogen peroxide begins to condense on surfaces, and it is designed to achieve a humidity level set prior to the start of the cycle [30]. This system is noncondensing VHP because the vapor stream is dried as it is returned to the generator [31]. It is virucidal, bactericidal, sporicidal, and active against mycobacteria including C. difficile spores, MRSA, and a wide range of nosocomial pathogens [32, 33]. Its long cycle times have made it difficult to use this system in health care facilities. It is efficient against fungi, viruses, MRSA, VRE, C. difficile, Klebsiella, Serratia, Mycobacterium tuberculosis, and Acinetobacter [34, 35].

*Dilute hydrogen peroxide (DHP)*. This technique uses water vapor and oxygen in the ambient air to continuously produce ozone-free hydrogen peroxide [36]. The environmental hydrogen peroxide produced is 0.02 ppm that is well below human safety thresholds. In fact, a level of 1 ppm is the max safety level of exposure according to the Occupational Safety and Health Administration and International Labour Organisation [37, 38]. DHP is active against a variety of viruses, bacteria, and fungi. It can be used during routine clinical practice in conjunction with established cleaning and decontamination methods. So, there are no restrictions on the use of a room for a period of time in practices.

*Surface disinfection via aerosol (SDVA)*. The device produces dry fog through a turbine at high speed that atomized and sprays disinfectant. Usually, H_2_O_2_ and hypochlorous acid (HOCl) are used as a disinfectant (Figures 1 and 2). The disinfectant is atomized into ultrafine droplets, blown into the air, and, after 10-30 min, settles on all surfaces; these disinfectant droplets quickly begin to take effect. The generator produces on average size 5 *μ* particles of disinfectant and ensures a slow and completely uniform sedimentation on each square of the treated premises with no humidity. The dry fog is displaced at 15 m thanks to the venturi effect. This nonwetting and noncorrosive fog can be used for all surfaces including electronic ones and is environmentally friendly. There are two stages for completing disinfection, spraying, and contact time. When the disinfection cycles are completed, it is required to open the windows for almost 10 min. So, the total time required for completing the cycle is 10-30 min. The H_2_O_2_ is nontoxic because it degrades in H_2_O and O_2_. Hypochlorous acid (HOCl) is a weak acid and has a virucidal power 300 times that of chlorine and is widely used for the decontamination of swimming pools. It is safe and used for nasal irrigation in patients affected by chronic sinusitis. A study showed a low (0.85%) concentration HOCl solution can be used as an effective nasal irrigation solution [39]. Hypochlorous acid (HOCl) has demonstrated broad-spectrum antimicrobial activity while being suitable for general use [40]. 20 to 200 ppm of HOCl solution resulted in ≥99.9% reduction of noravirus contagion on inanimate surfaces and aqueous suspensions [40], with low potential to damage treated surface materials [41]. The generator produces droplets of size ranging between 20 and 50 *μ*m. The HOCl fogs to concentrations ranging from 20 to 200 ppm and has virucidal effect against human norovirus [42]. Fogging is a mechanical action that produces small particles that can accelerate the interfacial mass transmission of chlorine gas. Low concentrations of hypochlorous acid (HOCl) have been demonstrated to exhibit both anti-influenza virus and antibacterial activity, but HOCl is also used to kill human rhinovirus (HRV) [42]. HOCl is considered by the FDA the agent that has the highest bactericidal activity against a broad range of microorganisms (US FDA, 2015) [43]. Avian influenza (H5N1) virus inactivation through fog applications of HOCl was achieved in 10 seconds [44]. HOCl has a temporary and gentle chlorine smell that dissipates rapidly.

UVC light (207–222 nm) is not visible to the human eye. Ultraviolet C radiation (UVC) emits light (207–222 nm) with efficient bacteria inactivating deliver-specific doses at different powers, for vegetative bacteria 12,000 *μ*Ws/cm^2^ and high power at 22,000–36,000 *μ*Ws/cm^2^ for spores [45, 46].

The UV light also inactivates drug-sensitive and multi-drug-resistant bacteria and viruses [47].

This technology is very limited because conventional UVC light sources are a human safety hazard, with a carcinogenic effect [48]. For this reason, the power of UVC light has been lowered to 2 mJ/cm^2^ and a recent study showed an efficiency when the lamps were positioned in public locations, reducing incidences of transmission of tuberculosis and influenza epidemics [49]. They are very efficient for the disinfection of health care environmental surfaces after manual cleaning has been performed. So, UVC irradiation treatments are effective for inactivating SARS-CoV. A continuous 30 min ultraviolet radiation is required to disinfect target surfaces and air [50].

There is a problem that natural and synthetic polymers are attacked by ultraviolet radiation, materials that make up many parts of a dentist chair, and other medical devices that include polypropylene.

Pulsed-xenon (PX-UV) systems emit high-intensity broad-spectrum UV irradiation in the 200–320 nm range [51] and are a means of quickly producing germicidal UV [51].

Usually, this is a portable device used in empty patient rooms because prolonged exposure to UV-C can cause eye and skin irritation. Fifteen minutes of PPX-UV exposure time can eliminate the pathogenic microorganisms [52] against 45 min required to clean a room with bleach [53].

Gaseous ozone is used for environmental disinfection [54]. It has antimicrobial and antiviral properties inclusive of Ebola although its mechanisms of action are not well understood [55, 56]. The device generates ozone and increases the ozone gas peaking at 20–25 ppm and includes ozone's known corrosive properties [20]. This technology is more efficient when there is low relative humidity [23]. It only takes 3-4 ppm to reduce all viruses and bacteria [57], but at 25 ppm, it is a disinfectant, while at 50+ ppm, it sterilizes surfaces. Ozone can damage the lungs when inhaled, a recent study showed in a rat model that increased methylation of the apelin promoter downstream of DNA damages the lungs, causing the development of pulmonary edema [58].

The generators are unable to elevate ozone levels near the required ppm range even in a small or average-sized room (<1-5 ppm). One to two hours of treatment are needed and 10-15 min of reentry after ventilation or open windows.

## 4. Discussion

During dentistry activity and the use of high-speed drills, droplets that are contaminated with the virus [59] can spread as far as two meters on to exposed surfaces [60] with environmental contamination and these remain infectious on workstation surfaces, medical instruments, etc. at room temperature for up to 9 days [61].

In fact, dental instruments such as rotating devices or ultrasonic devices use high-speed gas to drive the turbine to rotate at high speed and work with running water, and some dental procedures can cause coughing and, in any case, the patient breathes. The airborne droplets are of different dimensions and contain virus or bacteria pathogens which may survive on inanimate surfaces up to several months, and they may serve as a reservoir for cross-contamination with self-inoculation, as contaminated hands are a route for disseminating respiratory infections [62, 63].

In addition to the infected patients, there are the asymptomatic ones who can be negative to current health status investigations and/or the presence of risk factors for Covid-19 [64, 65]. For this reason, all patients must be treated during dental procedure as being Covid-19 positive. Hence, this is a timely topic, and dental clinics would be interested in the state of the art with respect to sanitization procedures. Several studies have found that hygiene quality management in the dental office may be problematic and surface microbial contamination has been found [66, 67]. All environment surfaces can become contaminated with infectious droplets from sprays of oral fluids or from touching them with contaminated fingers. The surfaces most frequently touched are drawer knobs, light handles, unit switches, dental radiograph equipment, reusable containers of dental materials, drawer handles, and dental chairside computers, and when these devices are touched, microbial agents can be transferred to other instruments [15]. General cleaning and disinfection with chemical or physical agents are recommended for device contact surfaces. It is very important to know material compatibility with physical or liquid chemical germicides. When wiping or scrubbing is used to remove microorganisms, any antimicrobial effect provided by the agent is reduced as there can still be a risk of creating another reservoir for microorganisms in the diluted solutions of the disinfectants themselves [68].

Disinfection of instruments and workstation surfaces against microbial contamination and inefficacy of environmental decontamination could be risk factors for cross-infection. Disinfection of surfaces is a method for reducing the risk of contact to viruses and interrupting their spread [69]. In dentistry, conventional manual disinfection of medical device surfaces is used, and this needs a two-stage disinfection procedure which includes surface rehydration followed by disinfection, for effective inactivation of bacteria and viruses on dry surfaces [70]. It is important to improve ventilation of health care spaces to dilute and clear out potentially infectious aerosols [10, 11]. Ventilation can reduce virus concentration in the air, limiting airborne transmission, but also the settling of viral particles, causing fomite transmission, for example, in influenza viruses [71]. The use of high ventilation rates during and after aerosol-generating procedures, such as high-speed drills, or piezosurgery [72–75] or between two patients has the potential to efficiently reduce circulating concentration of viral particles.

Environmental disinfection of the dental clinic is very important because the coronavirus can persist on inanimate surfaces like metal, glass, or plastic for up to 9 days, but fortunately, it is very sensitive to the action of disinfectants [61]. A recent correspondence in The New England Journal of Medicine showed that the stability of SARS-CoV-2 was like that of SARS-CoV-1 and was more stable on plastic and stainless steel than on copper and cardboard, and viable virus was detected up to 72 hours after application on these surfaces [76]. Different disinfectant agents were used against severe acute respiratory syndrome (SARS), Middle East Respiratory Syndrome (MERS), mouse hepatitis virus (MHV), canine coronavirus (CCV), and human coronavirus (HCoV) such as ethanol [77], 2-propanol [78], benzalkonium chloride [79], dodecyl dimethyl ammonium chloride [80, 81], chlorhexidine digluconate [80], sodium hypochlorite [82], hydrogen peroxide [83], formaldehyde [78], glutardialdehyde [82], and povidone-iodine [84]. The WHO recommends environmental cleaning and disinfection procedures which must be followed correctly. Benzalkonium chloride and chlorhexidine digluconate are not very effective or basically ineffective.

The most effective disinfectants are ethanol at strong concentration while sodium hypochlorite and hydrogen peroxide require a minimal concentration to be effective with a low impact on human health. Also, ethanol at 62 and 71% is similarly efficacious against coronavirus but can be used for small surfaces [85]. Ethanol has been widely used for the decontamination of hands based on 80% ethanol or 75% 2-propanol, and these are sufficiently efficacious [86].

For cleaning the workstation surfaces, sodium hypochlorite is suitable at a concentration of 0.05% with efficient and sufficient procedures [85] and when used at a concentration of 0.1%, it is effective in 1 min. Also, hydrogen peroxide is effective with a low concentration of 0.5% and an action time of 1 min. It is used for cleaning and disinfection implant drills because it preserves the drill structure after 50 cycles of decontamination [87–89].

Thorough decontamination and disinfection of all workstation surfaces in the hospital are very often difficult to achieve on multiple surfaces and complex equipment with wiping or scrubbing and require a lot of time.

For this reason, systems have been proposed, which offer the potential to improve the efficacy and reliability in hospital disinfection of environment and surfaces such as aerosolized hydrogen peroxide [18], hydrogen peroxide-producing systems [19], H_2_O_2_ vapor [20], hydrogen ultraviolet C light [21], pulsed xenon [22], and gaseous ozone [23].

There are differences between these systems in terms of their effectiveness, technological aspects, and microbiological efficacy. No data were found in the Guidelines for Infection Control in Dental Health-Care Settings 2003 and 2016. UV-C activity against viruses and bacteria is strongly influenced by distance and exposure times and has the most critical parameters; for this reason, a mobile ultraviolet-C device has been introduced [90]. A recent study showed that 6 min PX-UV disinfection is required to disinfect target surfaces and air, so it is fast and effective disinfection [91]. PX-UV disinfection is an effective agent for decontaminating the workroom. However, UV radiation may cause a significant degradation of synthetic polymers such as polystyrene which results in breaking the polymer chains [54].

The performance of different systems must be evaluated for use in dental practice. The UVC light and PX-UV systems are efficacious methods for decontamination of a room, but both systems attack synthetic polymer materials and many parts of dentist chairs and other medical devices can be damaged. The gaseous ozone requires a high concentration and in practice is very difficult to achieve without sealing the doors. So, the most interesting techniques for decontamination in clinical practice are VHP and aHP both of which use HP vapor or aerosol and are widely used for environmental decontamination in hospitals [92]. It is desirable that these techniques are also applied to dentistry.

Manual disinfection of work surfaces can result in poor disinfection of work stations with the risk of spreading pathogens from one surface to another [93]. However, there are many variables that influence the efficacy of the manual disinfection process such as distribution and contact time of the agent, which further limit the repeatability and reliance for an operator. For example, quarternary ammonium is an efficacious agent but when used with cotton or wipes containing substantial amounts of cellulose, the antimicrobial efficacy of the disinfectant may be reduced [94, 95]; therefore, it is recommended to use microfiber [96]. Another error is inappropriate overdilution of disinfectant solutions resulting in inappropriately low concentrations.

Outbreaks and rapid transmission of some viral diseases like rhinovirus, influenza, avian influenza, SARS, and infectious bronchitis, with their elevated morbidity and mortality rates, are generally attributed to infection via aerosol. Droplets produced during the use of high-speed handpieces and air/water syringes with the patient's saliva contaminate the air and floor, all work surface walls, and the objects that are nearby. Then, a no-touch or automatic disinfection approach to disinfection is needed to improve disinfection of surfaces in the dental clinic.

The major problem in clinical practice is that many enteric and respiratory viruses can be shed at great concentrations and contaminate and survive for long periods on environmental and medical device surfaces; this has been shown to play a role in their transmission [97]. HPV is a vapor-phase disinfection method. It is virucidal, including against influenza, and hence can be considered for the environment decontamination and disinfection of virus-contaminated surfaces in the dental clinic.

This technique is also very safe; in fact, it has also been used for the disinfection of N95 respirators with a residual level of H_2_O_2_ on the inner facial filter respirator at a very low level, 0.6 ppm at 2 hours and undetectable at 3 hours when the safety limit is actually lower, being <1 ppm [98]. Also, HOCI is a fast and simple technique that can be implemented in the dental clinic, since slightly acidic hypochlorous acid water has very fast and strong efficacy against pathogens [99].

Biosecurity programs have a critical role in the control of all infectious diseases. The main way to control and prevent those diseases that are airborne in the hospital or dental clinic is inactivation of infectious agents by spraying disinfectants in the air. HOCI is very popular for its broad and strong disinfection ability, demonstrating a very fast and strong efficacy against avian influenza and many viruses in a short contact time (5 sec), in vitro [44]. It has shown activity also against many bacteria and other microorganisms such as Staphylococcus aureus and Pseudomonas aeruginosa. Application of HOCI in low concentrations 20-200 ppm, by a spraying system with high turbine speed with the ability of producing aerosol particles (3-10 *μ*) inside dental clinics, is able to reduce the chances of aerogenic infection causing outbreaks and can limit virus transmission from one site to another. This powerful weapon is 100 percent safe for humans as it occurs naturally in our bodies. Neutrophils are white blood cells that are the first to arrive on site when an invading microorganism is detected. Neutrophils will chase down and engulf the pathogen through phagocytosis. Upon contact, neutrophils release a burst of bactericidal chemicals including its most effective oxidizing agent, HOCl. This inactivates the pathogen by destroying the cell membranes and proteins [100]. All the articles discussed in this review concern the control of infections of very resistant agents (such as norovirus, Ebola, methicillin-resistant Staphylococcus aureus, and C. difficile); for this reason, we can deduce that they are also active against influenza viruses which are much more sensitive to common disinfectants. Very few studies on dental clinics and the identified potential methods to achieve decontamination are detected in literature. So the decontamination technique that best suits the needs of the dental clinic is peroxide and hypochlorous which can be sprayed via a device at high turbine speed with the ability of producing small aerosol particles, recommendable also for their low cost.

These procedures do not replace the correct use of personal protective equipment [101, 102]. The lower the shed quantity (via the use of masks and safety glasses to limit shedding), the easiest it is to reach noninfectious doses after disinfection, and the lower the exposure dose, the lower the probability to get infected (via the use of masks to limit inoculation) [103, 104]. Although all dentistry procedures cannot be realized with a mask on the patient, it is important for the dentist to wear correctly one, in addition to colleagues entering the room, and patients in the waiting room for instance. We believe that no-touch methods augment manual cleaning but cannot replace it.

## 5. Conclusions

Dentists should consider the use of these disinfectants and no-touch decontamination technologies to improve disinfection of surfaces in dental clinics. In conclusion, manual cleaning and disinfection of environmental surfaces in health care facilities (daily and at patient discharge) are essential elements of infection prevention programs, especially during the SARS-CoV-2 pandemic.

## Figures and Tables

**Table 1:** Aerosolized hydrogen peroxide system (aHP) literature.

Aerosolized hydrogen peroxide systems (aHP)			
Author	Year	Design	Results
Chan et al. [24]	2011	Efficacy of hydrogen peroxide vapor decontamination on different surfaces of an Australian hospital, seeded with vancomycin-resistant *Enterococci (VRE)*	The 33.3% of the high-touch areas assessed had aerobic bacterial count below the detection limit post H_2_O_2_ decontamination, with the highest microbial density of ≤3 c.f.u./cm
Fu et al. [26]	2012	Efficacy and safety of hydrogen peroxide vapor (HPV) and aerosolized hydrogen peroxide against 10(6) methicillin-resistant *Staphylococcus aureus (MRSA), Clostridium difficile*, and *Acinetobacter baumannii*	The HPV system was safer, faster, and more effective for biological inactivation
Orlando et al. [27]	2008	Different concentrations (1, 2, and 4 mL/m^3^) of the hydrogen peroxide disinfectant were nebulized inside a 50 m^3^ experimental environment	The reduction of mean bacterial loading at concentrations of 1, 2, and 4 mL/m^3^ was 54.9%, 70.9%, and 86.9%
Holmdahl et al. [28]	2011	In vitro comparison of a hydrogen peroxide vapor (HPV) and aerosolized hydrogen peroxide (aHP)	ll BIs were inactivated for the 3 HPV tests, compared with only 10% in the first aHP test and 79% in the other 2 aHP tests

**Table 2:** H_2_O_2_ vapor heat-generated vapor system (aHP) literature.

H_2_O_2_ vapor heat-generated vapor			
Author	Year	Design	Results
Sk et al. [29]	2011	Effect on high strength AISI 4340 steel after exposure to vaporized hydrogen peroxide	No effects were produced for samples exposed to vapor hydrogen peroxide for concentrations up to 1000 ppm H_2_O_2_ and exposure times of 4.8h
Hall et al. [30]	2007	Effect of hydrogen peroxide vapor (HPV) disinfection on *M. tuberculosis* and *Geobacillus stearothermophilus*	Both groups were deactivated in all 10 locations following 90 min of HPV exposure
Otter et al. [31]	2013	No-touch automated room disinfection (NTD) system evaluation	NTD systems are a useful tool for infection prevention and control
Berrie et al. [32]	2011	Dried recombinant *adenovirus (Ad5GFP)* was tested before and after HPV exposure to determine the efficacy of hydrogen peroxide vapor HPV at inactivating adenovirus	HPV is effective for the inactivation of recombinant *adenovirus* and decontamination
Goyal et al. [33]	2010	Efficacy of hydrogen peroxide vapor (HPV) for the inactivation of *Feline calicivirus* (FCV)	The hydrogen peroxide resulted in a >3 log10 reduction in FCV infectivity and all but the 15 mL
Jeanes et al. [34]	2005	Hydrogen peroxide vapor (HPV) decontamination to eradicate MRSA environmental contamination in a surgical ward	Decontamination using HPV provides a rapid and cost-effective method for the eradication of environmental MRSA
Gopinath et al. [35]	2013	*NDM-1 Salmonella Senftenberg* (NDM-SS) drug resistance isolated in a patient. The environment was disinfected by hydrogen peroxide technology	Decontamination using hydrogen peroxide technology provides an effective method for NDM-1 Salmonella Senftenberg (NDM-SS)

**Table 3:** Dilute hydrogen peroxide (DHP) literature.

Dilute hydrogen peroxide (DHP)			
Author	Year	Design	Results
Oon et al. [36]	2011	Dilute hydrogen peroxide (DHP) in a critical care unit and measure the microbiological impact on surface contamination	Significant reduction in aerobic colony counts did not occur when the DHP was operating compared with baseline and control phases
OSHA guidelines [37]	2017	Samples are collected by drawing workplace air through two 25 mm quartz filters, coated with titanium oxysulfate, using personal sampling pumps	H_2_O_2_ evaporated off the cassette wall and reacted with the titanium oxysulfate-coated quartz filter
ILO guidelines [38]	2017	Harmful contamination of the air can be reached rather quickly on evaporation of this substance at 20°C	Decomposes under the influence of light on warming producing oxygen. Increase of fire hazard and is a strong oxidant. Attacks many organic substances such as textiles and paper

**Table 4:** Surface disinfection via aerosol (SDVA) literature.

Surface disinfection via aerosol (SDVA)			
Author	Year	Design	Results
Boyce et al. [92]	2016	No-touch technologies include aerosol and vaporized hydrogen peroxide, mobile devices that emit continuous ultraviolet (UV-C) light, a pulsed-xenon UV light system, and use of high-intensity narrow-spectrum (405 nm) light	Environmental departments should consider the use of newer disinfectants and no-touch decontamination technologies to improve disinfection of surfaces in health care

**Table 5:** HOCI generated fog (VHOCI) literature.

HOCI generated fog (VHOCI)			
Author	Year	Design	Results
Kim et al. [39]	2008	Human primary nasal epithelial cells treated with 3.5 ppm of hypochlorous acid for cell cytotoxicity	No cytotoxicity at 30 minutes or 2 hours after treatment with HOCl was recorded. More than 99% of bactericidal or fungicidal activity was noted for all species, except for Candida albicans
Park et al. [40]	2007	Efficacy of hypochlorous acid (HOCl) solution (HAS) to reduce NV in aqueous suspensions and inanimate carrier	Exposing virus-contaminated carriers of ceramic tile (porous) and stainless steel (nonporous) to 20 to 200 ppm of HOCl solution resulted in > or =99.9% (> or =3 log10) reductions of both infectivity and RNA titers of tested viruses within 10 min of exposure time
Russel et al. [41]	1999	Systematic review of sterilization methods, with uses and advantages outlined for each and valuation of disinfectants and their mechanisms of action with respect to current regulations	HOCI generated fog methods effective for the elimination or prevention/control of microbial growth
Yu et al. [42]	2011	Cells were infected with *human rhinovirus* for 24 hours and treated with HOCl three times, for 5 minutes each time, at 12-hour intervals	HOCl treatment significantly inhibited HRV-induced secretion of IL-6 and IL-8 and significantly reduced viral titer
Lister [43]	1952	The rate of decomposition of hypochlorous acid has been measured in an aqueous solution in the presence of much sodium hypochlorite	Values for the rate constants at different temperatures of all these reactions are given. Measurements are also reported on certain equilibria present in these solutions: the ionization of hypochlorous and chlorous acids, and the reaction
Hakim et al. [44]	2014	HOCl solutions containing 50, 100, and 200 ppm chlorine or their sprayed solutions were mixed with the virus with or without organic materials against a low pathogenic *avian influenza virus* (AIV), H7N1	In the indirect spray form, after 10 sec of spraying, the lids of the dishes were opened to expose the virus on rayon sheets to HOCl. In this form, the 200 ppm solution inactivated AIV within 10 min of contact, while 50 and 100 ppm could not inactivate the virus

**Table 6:** UVC light (207–222 nm) literature.

UVC light (207–222 nm)			
Author	Year	Design	Results
Boyce et al. [45]	2011	*Clostridium difficile* aerobic colony counts were calculated for each of 5 standardized high-touch surfaces in the rooms before and after UV light decontamination (UVLD)	The mobile UV-C light unit significantly reduced aerobic colony counts and C. difficile spores on contaminated surfaces in patient rooms
Nerandzic et al. [46]	2010	Cultures for *C. difficile*, methicillin-resistant *Staphylococcus aureus (MRSA)*, and *vancomycin-resistant Enterococcus (VRE)* were collected from commonly touched surfaces before and after the use of an automated ultraviolet radiation device	Efficient environmental disinfection technology that significantly reduces *C. difficile, VRE*, and *MRSA* contamination on commonly touched hospital surfaces
Conner-Kerr et al. [47]	1998	UV light (254 nm, 15.54 mW/cm^2^ output). Irradiation times were 0, 2, 5, 8, 15, 30, 45, 60, 90, or 120 seconds in killing antibiotic-resistant strains of *Staphylococcus aureus* and *Enterococcus faecalis* in vitro	Kill rates were 99.9 percent for the methicillin-resistant strain of *S. aureus (MRSA)* at 5, 8, 15, 30, 45, and 60 seconds and 100 percent at 90 and 120 seconds. Kill rates were 99.9 percent at 5, 8, 15, and 30 seconds for *vancomycin-resistant E. faecalis (VRE)* and 100 percent at 45, 60, 90, and 120 seconds
Setlow et al. [48]	1993	Irradiated groups of five 6-day-old fish with narrow wavelength bands at 302, 313, 365, 405, and 436 nm and scored the irradiated animals for melanomas 4 months later	The light energy absorbed in melanin is effective in inducing melanomas in this animal model and that, in natural sunlight, 90-95% of melanoma induction may be attributed to wavelengths >320 nm—the UV-A2 and visible spectral regions
Welch et al. [49]	2018	Far-UVC light (207-222 nm) efficiently inactivates bacteria without harm to exposed mammalian skin	Far-UVC efficiently inactivates aerosolized viruses, with a very low dose of 2 mJ/cm^2^ of 222 nm light inactivating >95% of aerosolized H1N1 influenza virus

**Table 7:** PX-UV disinfection system literature.

PX-UV systems			
Author	Year	Design	Results
Stibich et al. [51]	2011	The use of pulsed-xenon ultraviolet (PX-UV) room disinfection by sampling frequently touched surfaces in *vancomycin-resistant enterococci* (VRE) isolation rooms	The PX-UV system showed a statistically significant reduction in microbial load and eliminated VRE on sampled surfaces when using a 12-minute multiposition treatment cycle
Jinadatha et al. [52]	2014	Standard manual room cleaning to PPX-UV disinfection technology for MRSA and bacterial heterotrophic plate counts (HPC) on high-touch surfaces in patient rooms	PPX-UV technology appears to be superior to manual cleaning alone for MRSA and HPC. Incorporating 15 minutes of PPX-UV exposure time to current hospital room cleaning practice can improve the overall cleanliness of patient rooms with respect to selected microorganisms
Ghantoji et al. [53]	2015	High-touch surfaces in rooms previously occupied by *C. difficile*-infected patients were sampled after discharge but before and after cleaning using either bleach or nonbleach cleaning followed by 15 min of PX-UV treatment	After disinfection, the mean level of contamination for bleach was 0.71 c.f.u. (*P* = 0.1380), and 1.19 c.f.u. (*P* = 0.0017) for PX-UV disinfected rooms
de Groot et al. [90]	2019	UV-C exposure times and distance in killing *C. auris*, using strains from different countries	A maximal effect of *C. auris* killing was found after 30 minutes of UV-C exposure at 2 m. With half the time or twice the distance, the efficacy strongly diminished to ~10 and ~50 fold
Li et al. [91]	2020	Portable pulsed-xenon ultraviolet (PX-UV) machine on samples was taken from the surface of research tables	PX-UV disinfection also significantly reduced residual bacterial counts
Yousif and Haddad [54]	2013	UV radiation causes photooxidative degradation which results in breaking of the polymer chains, produces free radical, and reduces the molecular weight, causing deterioration of mechanical properties and leading to useless materials, after an unpredictable time	Free hydrogen radicals diffuse very easily through the polymer matrix and combine in pairs or abstract hydrogen atoms from polymer molecule

**Table 8:** Gaseous ozone disinfection literature.

Gaseous ozone			
Author	Year	Design	Results
Moat et al. [102]	2009	The efficacy of the approach using gaseous ozone for room sanitization was assessed	Application of the process in a 30 m^3^ room showed similar reductions in viable counts for *Clostridium difficile spores*, *Escherichia coli*, and *methicillin-resistant Staphylococcus aureus*
Hudson et al. [55]	2009	Develop a practical method of utilizing the known antiviral properties of ozone in a mobile apparatus that could be used to decontaminate rooms in health care facilities	All 12 viruses tested, on different hard and porous surfaces, and in the presence of biological fluids, could be inactivated by at least 3 log10, in the laboratory and in simulated field trials
Rowen [56]	2019	Ozone therapy, the most studied and least expensive to perform, is in itself a germicide, not an antibiotic, and improves several physiological parameters essential for infection defense	Very favorable responses to both bacterial and viral disease, inclusive of *Ebola*. Despite the lack of commercial profitability (not patentable), medicine would do well to revisit its preantibiotic era oxidation therapy roots, especially ozone in the current crisis
Hudson et al. [57]	2007	Ability of ozone gas to inactivate *norovirus* and its animal surrogate *Feline calicivirus (FCV)* in dried samples placed at various locations within a hotel room, a cruise liner cabin, and an office	QRT-PCR assays indicated similar decreases in both viral RNAs. Virus-containing samples dried onto hard surfaces (plastic, steel, and glass) and soft surfaces such as fabric, cotton, and carpet were equally vulnerable to the treatment
Miller et al. [58]	2018	Acute inhalation of ozone induces DNA methylation of apelin in the lungs and if a change in expression is related to altered DNA methylation in the lung	Ozone exposure reduced DNA cytosine-5-methyltransferase (DNMT) activity and Dnmt3a/b gene expression. Epigenetic modifications accompanied ozone-induced reduction of apelin expression and development of pulmonary edema
Ding et al. [103]	2019	Ozone disinfection of chlorine-resistant bacteria in drinking water	The ozone resistance of bacteria *Aeromonas jandaei < Vogesella perlucida < Pelomonas < Bacillus cereus < Aeromonas sobria* was lower than that of spores *Bacillus alvei < Lysinibacillus fusiformis < Bacillus cereus* at an ozone concentration of 1.5 mg/L. More than 99.9% of Bacillus cereus spores were inactivated by increasing ozone concentration and treatment duration

**Table 9:** Summary for each of the decontamination procedures for instance with the columns procedure, supply required, and threat to human health.

Decontamination procedure				
Procedure	Supply time	Deposition time	Room ventilation	Threat to human health
Aerosolized hydrogen peroxide	6 min/100 m^2^	1-2 h	15-30 min	Inhalation acute toxicity 1.93 mg/M^3^ Inhalation long-term toxicity 0.21 mg/M^3^
H_2_O_2_ vapor heat-generated vapor	15-50 min cycle	~130 min	15-20 min	Eye irritation, odor threshold
Surface disinfection via aerosol	15 minutes at 20°C	~2 h	~10 min	Eye irritation and mucosal tissue irritation
HOCI generated fog	10 min	30 min to 2 h	20-30 min	At prolonged exposure mild inflammatory reactions to mucosal tissues. At free chlorine low concentration pH 7.0 no toxicity
Dilute hydrogen peroxide	Continuous	~2 h	Not required	>3% hydrogen peroxide: mucosal tissue irritation, vomiting and diarrhea. Chronic inhalation: upper respiratory tract inflammation
UVC light (207–222 nm)	60-120 sec/30 m^2^	~	Not required	At direct exposure temporary damage and burns to the eyes, cornea, and potential carcinogen to the skin
PX-UV systems	12-30 min/30 m^2^	~	Not required	Skin and mucosal damage at prolonged exposure
Gaseous ozone	10-30 min/30 m^3^	~	10 min	Lungs damage, chest pain, coughing, shortness of breath, and throat irritation

## Abbreviations

SARS-CoV-2: Severe acute respiratory syndrome coronavirus
Covid-19: Coronavirus 19
UV: Ultraviolet light
HAS: Hypochlorous acid solution.
